# Twenty years of traditional and complementary medicine regulation and its impact in Malaysia: achievements and policy lessons

**DOI:** 10.1186/s12913-022-07497-2

**Published:** 2022-01-25

**Authors:** Ji-Eun Park, Junhyeok Yi, Ohmin Kwon

**Affiliations:** 1 Center for Global Collaboration, Korea Institute of Oriental Medicine, Daejeon, Republic of Korea; 2 Traditional Korean Medicine Policy Team, Korea Institute of Oriental Medicine, Daejeon, Republic of Korea; 3 Department of Data of Traditional Korean Medicine, Korea Institute of Oriental Medicine, Daejeon, Republic of Korea

**Keywords:** ASEAN, Malaysia, Traditional and Complementary Medicine, Integration, Institutionalization, WHO health systems framework

## Abstract

**Background:**

Many countries are trying to integrate traditional and complementary medicine (T&CM) into their health care systems. However, it is not easy to integrate T&CM within a given health care system. This study aims to draw policy outcomes and lessons from the case of Malaysia, which has been making efforts for over 20 years to integrate various types of T&CM into the national health care system (NHS).

**Methods:**

Documents were searched in major databases and websites using words such as Malaysia and T&CM, and additional documents were secured using snowballing techniques. Data were classified and organized according to the World Health Organization health systems framework.

**Results:**

Malaysia has focused on managing the safety and quality of T&CM, and to that end it has been institutionalized by enacting specialized laws rather than by applying existing medical law directly. Malaysia was able to institutionalize T&CM by adopting a step-by-step approach that considered the appropriateness of administrative policies and measures.

**Conclusions:**

Malaysia's experiences in implementing its T&CM policies will raise practical implications for countries struggling to integrate their existing T&CM into the NHS and utilize it for universal health coverage.

## Introduction

March 1, 2021 marks a historic milestone in the history of the Malaysian Health care system. Since that date, anyone wishing to practice in any recognized practice areas (RPAs) of Traditional and Complementary Medicine (T&CM) in Malaysia must register as a practitioner and hold a recognized qualification (Article 21) [1]. If anyone practices in an RPA without undergoing formal registration, they could face fines and/or imprisonment. Furthermore, no one will be able to practice in any area that is not designated as an RPA once the T&CM Act has been fully implemented. Over the last twenty years, the Malaysian government (MG) has developed policies to institutionalize and incorporate T&CM into the national health care system (NHS) and enforced regulatory measures for its practitioners and services. Consequently, it has reached the final stage of integrating T&CM into the NHS and is using it as a societal resource to provide universal health coverage (UHC). The policies and regulations represented by compulsory registration and professionalization have had an unprecedented impact on the reshaping of T&CM, and will bring about significant changes in the NHS of Malaysia, especially in terms of the relationship between modern medicine and T&CM and the provision of primary health care (PHC). The World Health Organization (WHO) recognizes the contribution of T&CM to UHC and encourages its member states to integrate and utilize T&CM for UHC [2, 3]. The WHO presents South Korea and China as examples of countries that have fully integrated T&CM into their NHS [3]. Both countries formally and successfully incorporated T&CM into their NHS in the mid-twentieth century, considerably earlier than any other countries. However, each country has institutionalized T&CM mainly in its own specific form of T&CM within a relatively homogeneous culture; while other T&CM modalities have been excluded from integration. Therefore, there will inevitably be a limit to how much any country can follow the experiences of, and draw lessons from, Korea or China with a single form of T&CM if countries start to integrate various types of T&CM characterized by cultural, political, or economic diversity.

Since Malaysia’s T&CM has a multiethnic and multicultural character, a wide variety of T&CM modalities coexist [4]. Furthermore, it is associated with a strong ethnic identity and is regarded as a cultural heritage. The MG is also taking a generous approach to various types of health care [5], and has been evaluated as an “in-process country” in which the integration of T&CM into the NHS is most actively under way [6]. The MG has steadily introduced diverse legislation on T&CM relatively early on, and has implemented or is planning to implement various measures for its institutionalization. Accordingly, when countries with cultural, political, or economic diversity make efforts to integrate various types of T&CM, it will be more pragmatic to refer to Malaysia rather than to Korea or China. This study delineates the status of T&CM, the dynamics of its institutionalization, and the challenges in Malaysia. It will allow us to consider issues and challenges that countries will face and learn policy implications if they set out to integrate T&CM into their NHS for UHC in other local settings and worldwide.

## Methods

The current study applies case study methodologies used in social science. This type of study carries out to grasp the social issues, event or phenomenon of interest on both historical and contemporary scenes [7]. It does not set hypotheses or estimate statistical relationship, but rather providing rich description of the case and draw the insights from phenomena in question. This study mainly relies on the literature review. Firstly, Databases such as PubMed, Google Scholar, and Malaysian Citation Index were searched with terms including ‘Malaysia’, ‘Traditional Medicine’, ‘Traditional and Complementary Medicine’, ‘Traditional Chinese Medicine’, ‘Traditional Malay Medicine’, ‘Traditional Indian Medicine’, and ‘ASEAN Traditional Medicines and Health Supplements Product Working Group (TMHS PWG)’. However, it was limited to obtain sufficient information through a conventional academic database search. Therefore, we referenced Malaysian government documents, research reports, legislative data, annual reports, academic papers in order to find T&CM data. We also reviewed data uploaded on the websites of the relevant organizations such as the T&CM Division (T&CMD) of the Ministry of Health Malaysia (MoH), the Malaysian National Pharmaceutical Regulatory Agency (NPRA), and the WHO.

Finally, by applying the snowball sampling technique [8], additional references were obtained from the previously collected data. The data were classified into five factors, i.e. governance (legislation, administrative organizations), human resources (education, practitioners), finance, services (utilization, provision, quality management), treatment tools (herbs, medicines, devices), thus reorganizing the WHO’s conceptual framework for health care systems [9]

## Results

### Governance

**Fig. 1:**
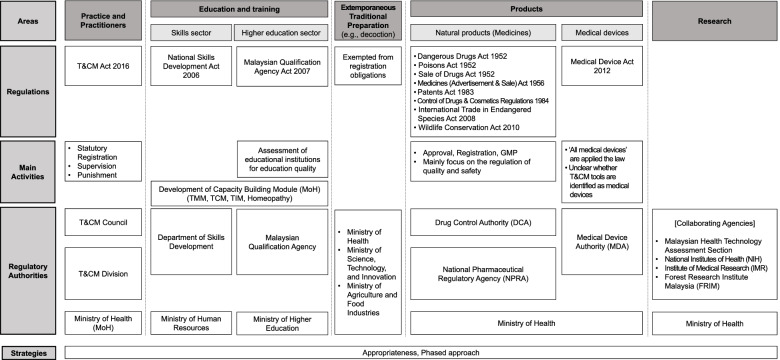
Malaysia’s T&CM sector-specific departments system (recited and edited) [4, 14]

The MoH has designated seven types of T&CM: Traditional Malay Medicine (TMM), Traditional Chinese Medicine (TCM), Traditional Indian Medicine (TIM), Homeopathy, Chiropractic, Osteopathy, and Islamic Medical Practice (IMP) as RPAs in Malaysia (Table 1) [1, 10]. The MG initiated the integration of T&CM into the NHS via the professionalization of practices and practitioners, which were previously entrusted only to a laissez-faire market mechanism. This shows an awareness of the growing demand for and supply of T&CM in the unregulated market and the increasing influence of T&CM on public health. The government has engaged with T&CM through administrative measures such as legislation, policies, and guidelines. Currently, the MoH is responsible for overall T&CM; the Ministry of Human Resources and Ministry of Higher Education for education and the training of practitioners; the Ministry of Agriculture and Food Industries (MAFI) for herb management; the NPRA for the supervision of T&CM products; and the Medical Device Authority (MDA) for regulation of the medical devices (Fig. 1) [4, 14]

**Table 1:** Brief descriptions on each T&CM in Malaysia

Recognized Practice Area	Descriptions
Traditional Malay Medicine	Knowledge and practices are indigenous to the Malay culture deal with aspects of health and healing. It takes a holistic approach based on physical and spiritual elements [10]
Traditional Chinese Medicine	Based on Chinese culture, knowledge and practices of valuable long-term experience in understanding life, maintaining health, and overcoming the disease. It uses various psychological and/or physical approaches and herbal products to address health problems. [11]
Traditional Indian Medicine	A group of certain of India's ancient indigenous medical approaches originated from two ancient treatises. Malaysia government recognize Ayurveda (science of life), Siddha (perfection of heavenly bliss), Unani, Yoga, and naturopathy as Traditional Indian Medicine. [12]
Homeopathy	The therapeutic system of medicine premised on the Similarity—“like cures like”—implies that substances possibly causing diseases are used as medicines to treat similar patterns of illness experienced by patients. [10]
Chiropractic	Focus on the relationship between bodily structure (primarily the spine) and function; and how that relationship affects the preservation and restoration of health. The manual treatment methods used by chiropractors range from stretching and sustained pressure to specific joint manipulation, reducing pain and disability, and promoting rehabilitation [11]
Osteopathy	An approach of detecting, treating, and preventing health problems by stretching, moving, and massaging muscles and joints. The principle is that a person's well-being relies on their bones, muscles, ligaments, and connective tissue functioning smoothly together [13]
Islamic Medical Practice	Therapeutic approach to treating physical and spiritual sickness by Muslims. Practitioners are skilled in treating using the verses of the Qur'an or hadith, or the practice of salaf al-soleh, ulamak muktabar, or all at once and using the techniques or materials that are allowed by shariah [10]

#### T&CM policy, governing organization, and administrative measures

The MG has officially recognized T&CM as a part of health care that is directly connected to public health, and accordingly initiates governmental interventions to secure the quality, safety, and effectiveness of T&CM.

In 1996, the MG formed, for the first time, a unit to take charge of T&CM affairs in the MoH and embarked on the management of T&CM. In 1998, a joint public–private T&CM Standing Committee was formed to advise on government policies for developing and regulating T&CM. In 2001, the government expressed its determination to promote and regulate T&CM by publishing the National Policy on Traditional/Complementary Medicine. In the Policy [15]*,* the MG declared the institutionalization, professionalization, and integration of T&CM into the NHS in the name of quality and safety. In 2004, the T&CM Unit was raised to the status of a T&CMD, which went on to play a leading role in drafting and enforcing the T&CM Act and designing a T&CM development plan. The T&CMD is composed of the following five sections and has about fifty staff: Policy and Development; Management and Training; T&CM Practice; Inspectorate and Enforcement; and T&CM Council [16].

Since its establishment, the T&CMD has continuously carried out specific and practical promotions or regulatory measures that have had substantial impacts. In 2006, T&CM services began to be provided at MoH hospitals. In 2008, the first drafting of a T&CM bill was completed, and the voluntary registration of T&CM practitioners took off. In 2010, regional branches in charge of T&CM affairs began to be established at the state level; and in 2012, government T&CM services were extended to the PHC level [4, 17].

Since the enactment of the T&CM Act in 2013, T&CM has undergone dramatic changes. The Act explicitly stipulates statutory and mandatory registration, required qualifications, the disciplinary proceedings of punishments, the enforcement of stop orders and closure orders, and search and seizure, etc. Since 2016, by enforcing the Act clause by clause, the MG has adopted a “phased approach” that promotes the integration and institutionalization of T&CM by stages. It also emphasizes “appropriateness” in developing regulations and health care models [4]

In 2018, the MG once again made a breakthrough with the completion of a ten-year initiative (*Traditional and Complementary Medicine Blueprint 2018–2027*), which lays out the regulatory integration and economic development of T&CM [4]. The T&CMD evaluates that forty-seven out of seventy-one action plans began to be implemented in 2019 [17], and that fifty-five action plans were initiated and eight completed in 2020 [18]. If the strategic objectives and action plans proposed by the *Blueprint* are fully accomplished, T&CM services are anticipated to become a genuine, full-scale health care profession, and practitioners to become fully qualified professionals, by 2027. As such, they will be subject to statutory regulation and formal management substantially equivalent to conventional medicine (Table 2).

**Table 2:** History of the institutionalization of traditional and complementary medicine in Malaysia

Year	Governance / Organizations	Regulation/Policy	Legislation	Practices/Services
1987		Completion of position paper on the need of research on T&CM		
1992		Registration of traditional medicines (products)		
1996	Formation of the T&CM Unit (Post-cabinet decision)			
1998	Formation of the Standing Committee on T&CM composed of governmental officers and representatives of T&CM bodies			
1999	Establishment of five T&CM umbrella bodies appointed by the MoH	Enforcement of GMP requirement for traditional medicines manufacturers Commencement of the licensing of T&CM manufacturers and importers		
2000	Establishment of the Herbal Medicine Research Center (HMRC) at the Institute of Medical Research (IMR)			
2001		Launch of the National Policy on T&CM		
2002	Establishment of the National Committee for R&D on Herbal Medicines		Formation of a working group to draft a bill on T&CM	
2003	Establishment of the National Institute for Natural Products, Vaccines & Biologicals· Launch of the prototype of the GlobinMed [web portal]			
2004	Establishment of the T&CMD			
2007	Expansion of the T&CMD to include the Inspectorate & Enforcement Section tasked with coordinating surveillance activities related to T&CM practices Official launch of the Globin Med	Revision of the National Policy on T&CM	Final stage of the drafting of the T&CM bill	Initial establishment of T&CM units (practices) at government hospitals
2008		Start of voluntary registration of T&CM practitioners through an online registration platform Start of enforcement activities on T&CM premises by the Inspectorate and Enforcement Section, T&CMD	Finalization of the T&CM bill	
2009				Publication of the first practice guidelines on T&CM practice
2010	Establishment of first T&CM branches at the state level to carry out T&CM administration			· Publication of first good practice guidelines · Institutions of higher education began to offer T&CM education accredited by the MQA
2013			Publication the T&CM Act	
2015		Start of engagements and discussions with stakeholders to develop the T&CM BluepriFnt Appointment of eight practitioner bodies for self-regulation and registration		
2016			Enforcement of the T&CM Act, replacing the T&CM Act 2013	Publication of Advertisement Guideline for T&CM Practitioner
2017	Establishment of the T&CM Council to oversee T&CM services and facilitate determination of relevant matters (**First phase** in the enforcement of the Act)		T&CM Order 2017 (Recognized Practice Areas) took effect T&CM Order 2020 (Designation of Practitioner Body) is deemed to have come into operation on 2017	
2018		Launch of the T&CM Blueprint Introduction of the TPC Transformation Plan to transform the TPC service and expand it from all related government hospitals to the PHC settings level		Publication of Consumer Guidelines on the Proper Use of T&CM Services and guidelines for the Evaluation of T&CM Practices
2021		Compulsory registration of T&CM practitioners with the Council (**Second phase**) [1 Mar 2021] Reception of applications for the registration of T&CM practitioners in Recognized Practice Areas [15 Mar 2021]		
March 2021 – February 2024		**Transitional period** for practitioners who lack a recognized qualification but have skills and experience in a particular practice area		
2024			Full-blown implementation of the T&CM Act 2016 **(Final phase)**	

#### Legislation for T&CM

The main gist of the T&CM Act − enforced in 2016 − is to guarantee the quality and safety of T&CM [1]. The Act covers the overall regulations on the management and supervision of T&CM services and practitioners, and contains the following provisions: specification of the RPAs; the designation and revocation of T&CM bodies; the self-regulatory framework; practitioners’ certifications and the management of educational institutions; the compulsory registration of T&CM practitioners and their cancellation; and the issuance of certificates to practitioners. Once the relevant clauses have been enforced, the Act will prohibit any practices that do not fall under the category of RPAs [1].

The Act imposes various qualifying requirements and obligations upon T&CM practitioners for the purpose of quality assurance, while granting them a monopolistic privilege and imposing accompanying duties similar to those of medical practitioners. Furthermore, its clauses broadly cover diverse matters ranging from medical malpractice and patient rights to the supervision and punishment of professional misconduct. In addition, it accords to the government the discretionary power to appoint the date of entry into force of a particular provision and to issue administrative orders.

### Human Resources

#### Education and training: improving the quality of education through regulation

As a substantial percentage of Malaysia’s T&CM practitioners provide T&CM services without having received any systematic education or training, the government has strengthened quality control by introducing various regulations on educational requirements, the design of a standard curriculum, and mandatory certification at the minimum level [10].

T&CM education in Malaysia is divided into two tracks—academic and skills education—the main goal of which is the quality control of institutions that provide academic education. As the universities that run T&CM courses must pass an assessment by the Malaysian Education Qualification Agency (MQA), T&CM educational institutions are struggling to meet the education standards specified for each type of T&CM and to overcome the challenges associated with a lack of the resources required to ensure the quality of education. Moreover, the T&CM Act stipulates stronger quality assurance of practitioners by requiring them to complete a residency of at least one year after completing the regular curriculum [1]. Although this provision has not yet been implemented, it will enter into force on the date appointed by the MoH. As of March 2021, among the private educational institutions that have operated the T&CM curriculum for RPAs, only four RPA—TMM, TCM, Homeopathy, and Chiropractic—have been certified by MQA [19]. Currently, eleven private universities and institutions provide seventeen accredited T&CM educational programs, while none of the public institutions provide T&CM education. Additionally, capacity-building courses in the fields of TMM, TCM, TIM, and homeopathy have been instituted for Malaysian practitioners who have not yet completed any formal training courses but who have acquired adequate practical experience, allowing them to qualify for registration [20, 21].

The TCM, under the strong influence of TCM in China, has established the most systematic education system among the seven types of T&CM. Currently, six of the eleven T&CM accredited universities provide TCM education; seven private universities have a TCM curriculum in Malaysia [19, 22].

#### Practitioners: moving from self-regulation towards statutory professionalization

T&CM practitioners make up a significant proportion of the health care workforce in Malaysia. However, the official number of practitioners has apparently begun to decrease since the introduction of formal registration. The MG formerly encouraged T&CM practitioners to voluntarily register with the MoH and promoted practice quality by allowing self-regulation by each professional organization [23]. Since the T&CM Act entered into force, the regulations have been gradually strengthened, including the switch to a system whereby licenses are only issued to practitioners who have received a certain level of training and a certification (Table 3) [24, 25]. In particular, from April 1, 2024, after the three-year transitional period of statutory registration, unregistered practitioners will not be able to provide T&CM services in any RPA. Furthermore, more and more practice areas will be prescribed as RPAs, and “no one shall have the right to practice in any practice area which is not an RPA” if the MG fixes a date for implementation of the relevant provision of the Act [1]. As of 2018, the number of T&CM practitioners who had registered voluntarily stood at 16,162, equivalent to 26.4% of the total number of medical doctors (61,158) [26]. In 2016, T&CM practitioners, TCM practitioners accounted for the largest share at 44.9%, followed by IMP practitioners (33%) and TMM practitioners (12%) [27]. The decrease in the T&CM workforce is remarkable among foreign practitioners, and this decline is expected to continue for the time being due to the tightened regulations [27].

**Table 3:** Qualifications of registered practitioners and designated bodies in Malaysia (recited and edited) [24, 25]

Recognized Practice Area	Subarea/Subfield	Qualification	Existence of Capacity Building Module^a^	Designated Practitioner Bodies
Traditional Malay Medicine	Malay herbs	Not available	O	Federation of Traditional Malay Medicine Practitioners Associations of Malaysia
	Malay massage	Level 4 (diploma): Therapeutic Massage and Care Advanced Diploma in Malay Massage from the Sultan Salahuddin Abdul Aziz Shah Polytechnic (2010–2012)		
	Malay cupping	Level 3 (certificate): Wind Cupping Therapy		
	Postnatal care	Level 4 (diploma): Mama Care Post-Natal		
Traditional Chinese Medicine	Chinese herbs	a) Bachelor’s Degree in TCM or equivalent; or b) Bachelor’s Degree in Acupuncture, Moxibustion and Tuina or equivalent; or c) Diploma in TCM, graduation from one of 15 listed local TCM colleges	O	Malaysian Chinese Medical Association Federation of Chinese Physicians and Medicine Dealers Associations of Malay Federation of Chinese Physicians and Acupuncturists Associations of Malaysia
	Acupuncture and Moxibustion			
	Chinese cupping			
	Tuina			
Traditional Indian Medicine^b^	Ayurveda	Bachelor’s Degree in Ayurveda or equivalent	O	Malaysian Association of Traditional Indian Medicine
	Siddha	Bachelor’s Degree in Siddha or equivalent	O	
	Unani	Bachelor’s Degree in Unani or equivalent	X	
	Yoga and naturopathy	Bachelor’s Degree in Yoga and Naturopathy or equivalent	X	
Homeopathy	-	Bachelor’s Degree in Homeopathy or equivalent	O	Malaysian Medical Homeopathic Council
Chiropractic	-	Bachelor’s Degree in Chiropractic or equivalent	X	Federation of Complementary and Natural Medical Association, Malaysia
Osteopathy^b^	-	Bachelor’s Degree in Osteopathy or equivalent	X	
Islamic Medical Practice^b^	-	Level 4 (diploma): Ruqyah Healing	X	Malaysian Islamic Medical, Medical and Welfare Association

Level 3: Malaysian skills certificate

Level 4: Malaysian skills diploma

Equivalent: As recognized by the Council

^a^ Module developed by the Ministry of Health to help local practitioners who lack basic registrable qualifications but who possess years of practical experience to register as T&CM practitioners under the T&CM Act 2016

^b^ No accredited institutions by the Malaysian Qualifications Agency for higher education programs

These regulations are expected to raise the quality and status of T&CM practitioners, who will be recognized as independent professionals above a certain level. However, the level of occupational recognition as a profession and the timing of such an achievement are likely to differ for each T&CM.

### Finance

#### Spending on T&CM

Expenditure on T&CM as a proportion of total health expenditure is rather low, despite being widely used in Malaysia [28]. In the public sector, T&CM services are provided only in fifteen government hospitals as of 2020, and most of the T&CM services are provided by almost private institutions [18]. Although the magnitude of T&CM OOP has more than doubled over the past decade (2009–2019), the proportion of all OOP has remained around 3% [29]. In 2019, about 93.2% of the out-of-pocket payment for health care in Malaysia was spent on modern medicine, and only 3.59% (180 million USD) was spent on T&CM in 2019 [29]. The cost of T&CM services provided in MoH hospitals is covered with government subsidies, while T&CM services in the private sector must be fully paid for by the patients themselves [30]. Since T&CM spending is concentrated on the private sector, it is difficult to estimate its exact figure due to the lack of relevant data. Recently, a project to collect data on the basic costs, including a survey to calculate the appropriate price of T&CM services, is currently under way [31].

### Services

#### Service utilization: common but highly dependent on the private sector

The utilization of T&CM services is common in Malaysia, and most of them are provided in private institutions. According to Peltzer et al., Malaysia’s T&CM utilization rate is 55.6%, the highest among ASEAN countries [32]. Other studies also report that 80.2% of patients use T&CM services [33], and the utilization by type of T&CM shows a similar percentage with regard to ethnic groups. TMM users, reportedly the largest group, account for 52%, followed by TCM users at 20%, and complementary therapy users at 6.2% [34]. T&CM is mainly used to alleviate pain caused by musculoskeletal diseases (64.3%) and nervous system diseases (12.1%) [35], following a similar trend observed in other countries [36].

#### T&CM services in public health care facilities and extension to the PHC level

A pilot project to provide T&CM services was launched in MoH hospitals in 2006 as part of a policy to integrate T&CM into the NHS. The MG has expanded the provision of T&CM services at the hospital level and extended them to the PHC level. As of 2020, fifteen MoH hospitals were providing a total of six T&CM practices in eighteen indications. Among the T&CM services covered, acupuncture accounts for the largest share with 37,989 cases (64.2%), followed by traditional Malay massage (18.6%), herbal therapy as an adjunct treatment for cancer (11.6%), External Basti therapy (3.2%), Varmam therapy (1.8%), and Shirodhara (0.6%) in 2019 [18].

The MG has also made steady attempts to integrate T&CM services into the PHC sector. Traditional Postnatal Care (TPC), for example, began to be provided to mothers by private practitioners under the pilot project conducted at the clinic level in Johor State in 2012. The mothers who participated in the TPC project reported a high level of satisfaction. Finally, TPC services were transferred to local PHC clinics from the MoH hospitals in 2018 in accordance with the TPC Transformation Plan. In 2020, TPC began to be provided in fifteen rural clinics and eighty-nine health clinics in fifteen states (Table 4) [17, 18]. This represents the first government attempt to expand the role of T&CM at the PHC level in Malaysia, and is in line with the WHO’s conviction that “Traditional medicine can contribute to strengthening primary health care” [37].

**Table 4:** History of transition of traditional prenatal care services to the primary health care level

Year	Main Contents
2006	Introduction of Traditional Prenatal Care (TPC) services at selected *government hospitals*
2012	Introduction of TPC services *for primary care* in the state of Johore (pilot project)
2014	Expansion of TPC services to the state of Kelantan (pilot project)
2015	Kick-off TPC at the primary health care level in KK Meranti, Kelantan
2016	Decision to expand TPC across the country and to all districts in Kelantan
2018	Introduction of the TPC Transformation Plan to transform TPC service and expand it from all related government hospitals to the *PHC* level; to be administered at clients’ homes at their own expense by private T&CM practitioners. Cessation of TPC services in government hospitals (transferred to the PHC level)
2019	Implementation of the TPC services Transformation Plan (Phase 2). Extension of TPC services to 8 states
2020	Provision of TPC in 15 rural clinics and 89 health clinics in 15 states. First national level T&CM TPC technical meeting

#### Management of service quality

The MG manages service quality in terms of practices, practitioners, and users. First, safe and good quality practices are encouraged by the distribution of (good) practice guidelines (PGs) [38, 39], the provision of direct education to practitioners by the [27], and the periodic registration of practitioners. Since 2007, the MoH has published and revised three good PGs and general PGs for eleven therapies, basically fostering self-regulation by T&CM practitioners. These activities show that the government undertook sincere efforts to secure the standardization and safety of T&CM practices very early on.

Second, measures for the supervision and punishment of T&CM providers have been put in place. Under the T&CM Act, the MoH may appoint officials with the authority to issue orders for the suspension or closure of T&CM services and premises once the relevant provisions take effect [1].

Third, it is preparing a mechanism that allow T&CM users to complain or file for a dispute resolution. The MoH annually publishes the results of complaint by users by type of T&CM. This mechanism is stipulated in the T&CM Act, and the MoH has shown a very high level of responsiveness with regard to consumers, notifying persons who file a complaint, responding the receipt of their report within 24 h [18, 30].

It is noteworthy that the government has shown its commitment and determination to ensure that the public receive safe and good quality T&CM services by strengthening the obligations of T&CM practitioners and the rights of users simultaneously. Once these measures have settled, the quality of T&CM practices is expected to improve further.

### Therapeutic apparatus

#### (Raw) Herbs: grey area

Medicinal herbs are used both as a self-cure by patients and as a professional remedy by practitioners. However, they fall within a rather grey area of ​​regulation in Malaysia. Fifty-six percent of Malaysian women use herbs, which are consumed in the form of raw herbs (25.1%), medicines and health supplements (17.2%), and others (13.2%) [40]. From the pharmaceutical point of view, herbs are classified into two forms: extemporaneous traditional preparations, which are directly prescribed and dispensed as raw and/or dried medicinal herbs by T&CM practitioners; and herbal products, which are processed and distributed through pharmaceutical companies for treatment purposes. While the former are exempt from the registration obligation, the latter are subject to GMP requirements, approval, and registration regulations, similar to those for synthetic drugs [41, 42]. Therefore, herbal therapies directly prescribed by T&CM practitioners still lie outside the scope of public administration.

In terms of safety and efficacy, they need − in principle − to be regulated throughout the entire value chain of T&CM medicines, i.e. herbal cultivation, collection, processing, manufacturing, and development. There is also growing recognition of the need to regulate the herbal value chain; however, what, how, and to what degree it should be regulated still remains unclear inside the government. The governance of herbal affairs is scattered across the MAFI, the Ministry of Science, Technology, and Innovation, and the NPRA. As such, this lack of herbal policy coordination is contributing to a corresponding lack of appropriate regulation of the value chain [43].

#### T&CM products (medicines): the large gap between regulation and the market

T&CM medicines*—*in the form of finished products— are supervised by the NPRA across the entire process encompassing registration, manufacturing, importing, distribution, prescription, and post-marketing surveillance. Practically speaking, however, the quality of T&CM medicines is not ensured in the market. This might be attributed both to the existence of a market mechanism designed to evade regulation and to the misuse of loopholes in the regulatory system.

A social consensus was reached early on that Malaysia should regulate T&CM products and practitioners separately [44]. In 1992, mandatory registration was introduced for herbal products, and from 1999 T&CM medicines manufacturers were obligated to comply with the GMP [45]. The NPRA manages T&CM products by categorizing them into ‘health supplements’, ‘natural products’ (NPs) (traditional and homeopathic medicines), and ‘NPs with a therapeutic claim’. As for NPs, like synthetic drugs, they fall within the scope of governmental regulation in three key aspects: efficacy, safety, and quality. Practically, however, the immediate goal of regulation centers on ensuring quality and safety, with efficacy issues placed lower down the agenda [4, 46]. To tackle the efficacy issues of T&CM medicines, the government created a new ‘NPs with a therapeutic claim’ registration track that can be applied to T&CM medicines with proven efficacy, but no T&CM medicines have met the requirements as yet. The NPs track only requires historical evidence of efficacy from pharmacopeia or traditional medical books when filing an application for registration to the NPRA, and does not require the robust clinical studies that are essential for synthetic drugs. Despite these eased regulations, manufacturers of T&CM medicines prefer to register their products as health supplements to circumvent the requirement to provide evidence of efficacy [47]. T&CM medicines account for about half of the products registered annually to the NPRA. For instance, T&CM medicines accounted for 52% (12,208 items) of all the products registered in 2019 [48]. Two-thirds of licensed pharmaceutical manufacturers produce T&CM medicines (173 locations, including health supplement manufacturers). This suggests that small manufacturers, motivated by the less strict registration standards, could easily enter the market for T&CM medicines; however, T&CM products are often recalled as they do not meet the safety and quality standards [49] and complaints in NPs and health supplements also are continuously reported [50].

Although the regulation of T&CM products commenced earlier than that of practitioners, it has not gone further. Thus, the MoH have concluded that an appropriate regulatory mechanism for T&CM medicines needs to be designed [4]. Considerable policy efforts and time should be invested to achieve a certain level of quality for T&CM medicines and expand the regulatory scope to cover the efficacy issue.

#### Medical devices: unclear

The regulatory mechanism for medical devices became full-fledged in Malaysia with the enactment of the Medical Device Authority Act [51] and the Medical Device Act [52] in 2012. It was introduced later than the regulatory mechanisms for other elements of the health care system, such as practices and medicinal products. According to these laws, ‘all medical devices’ are regulated across the entire process of their production, importation, use, and disposal. The scope of ‘all medical devices’ is interpreted to cover medical devices used for T&CM, such as acupuncture, cups for cupping, and vessels. However, it is unclear yet whether these treatment tools have been identified by the MG as medical devices requiring safety considerations.

## Discussion

This study aims to analyze the status of T&CM, the process of its institutionalization, and the challenges in Malaysia, and to derive implications for countries that are considering the integration of T&CM services into their NHS and utilize them for UHC.

The MG aims to institutionalize T&CM into the NHS by adopting a phased approach. The MG has instituted a specialized law that reflects the situation of T&CM rather than applying the existing law, which is customized for medical affairs. Malaysia has achieved a considerable level of T&CM institutionalization in terms of legislation, administrative measures and regulations, and the establishment of the necessary organizations. In particular, enforcing the T&CM Act has laid the groundwork for a major leap forward Malaysia’s T&CM—which was considered to be in a state of “structured chaos” caused by “the lack of any appropriate legislation”—to a “structured order” [53]. The MoH’s active administrative drive and persistent engagements have might played a pivotal role in materializing the T&CM Act’s potential with the formation of T&CM governance, professionalization, institutionalization, and final integration into the NHS. Timely legislation and the government’s strong policy commitment are considered to have exerted synergistic effects on the regulation and development of T&CM in Malaysia. Thus, the WHO evaluates Malaysia’s success as an exemplary model for the institutionalization of T&CM and its integration into the NHS for UHC [54]. The MG has employed a realistic approach that prioritizes the issues of quality and safety while leaving effectiveness for the future agenda in terms of policy planning and implementation. It has adopted the evolutionary concept of ‘appropriateness’—for example, appropriate regulation— and a ‘phased approach’, taking into account the current status of T&CM industries over time and place, rather than applying a ‘one-size-fits-all’ policy or a ‘one-policy-fits-all’ stance.

It is worth noting that the MG, represented by the MoH, has maintained a strong and consistent policy of integrating T&CM through professionalization and regulation over twenty years. As a governing agency of the government’s policy and will, the MoH set up the T&CMD, commissioned T&CM affairs, and kept the T&CMD stable for a long period, in order to coordinate the different interests of diverse stakeholders. During that period, officials of the T&CMD were assumed to be more knowledgeable and experienced in terms of policy design, implementation, and stakeholder negotiation. For example, the IMP entered the RPA just before the tabling of the T&CM Act at the National Assembly [55], the drafting of which required more than a decade of policy commitment. This shows that countries which are planning the integration of T&CM need to reach a social consensus based on a strong policy will and long engagements with T&CM stakeholders situated in different milieus, where various types of T&CM coexist.

T&CM is generally known to play its own role in the private PHC sector and remains outside the regulation. For this reason, the WHO aims to promote T&CM as an essential resource for UHC in the PHC, by ensuring that T&CM practices are backed up with evidence of their quality and safety [37, 56]. The MG has been pushing for the adoption of T&CM practices—with proven quality and safety—at government health care facilities. Therefore, a health care program designed by the T&CMD was implemented in local MoH offices.

When a country tackles the issues of how and where to locate T&CM at the PHC, the TPC program could be a good example of the role of T&CM in achieving UHC. TPC program was initiated as a health care program utilizing a particular T&CM practice at the governmental level and is now on course for transfer to the local primary level for expanded access. The regulation of T&CM products, including medicines and devices, remains in a grey area. One of the main reasons for this is that T&CM medicines are regarded as lying somewhere between food and drug products. Thus, difficulty in regulating such products is an issue that should be globally addressed, rather than a local challenge remaining unresolved in Malaysia alone. The use of T&CM products is often inextricably linked to cultural factors as well. Therefore, it is difficult to find a ‘one-policy-fits-all’ solution due to the great diversity of products in terms of origin and form. T&CM in Malaysia, in particular, is mainly characterized by manual techniques, while the utilization of herbal medicines or therapeutic tools is limited to specific modalities such as TCM. Thus, Malaysia’s T&CM regulation is focused on practices and practitioners, and T&CM products remain low on the list of policy priorities. It is also necessary to point out the limitations of the study. First, the current study dealt with the unique case of Malaysia—in progress—that integrates various modalities of T&CM in a country. Accordingly, few scientific studies have not been conducted yet, and many of the data in the analysis were sourced from government documents. In addition, as data in the study were analyzed from a descriptive perspective without setting a specific hypothesis, further studies are necessarily analyzed. For example, the dynamic process and consequences of policy change could be explored through qualitative research such as interviewing various stakeholders.

## Conclusions

The MG faces T&CM policy challenges that are shared globally, particularly in ASEAN countries. Nevertheless, Malaysia is an uncommon country in that it has institutionalized multiple forms of T&CM to a considerable extent, not only administratively and legally, but also socially. It places higher policy priority on the quality and safety of T&CM, and aims to achieve its goal by professionalizing and integrating practices and practitioners into the NHS. Malaysia’s achievements and experiences would provide valuable lessons and implications for countries that are planning to expedite the integration of T&CM into the NHS for UHC. It would not be an easy task, and might take a long time.

## Data Availability

All data are publicly available.

## Abbreviations

GMP: Good Manufacturing Practice
IMP: Islamic Medical Practice
MAFI: Ministry of Agriculture and Food Industries
MG: Malaysian government
MoH: Ministry of Health Malaysia
MQA: Malaysian Education Qualification Agency
NHS: National health care system
NPRA: Malaysian National Pharmaceutical Regulatory Agency
NPs: Natural products
PGs: Practice guidelines
PHC: Primary health care
RPAs: Recognized practice areas
T&CM: Traditional and complementary medicine
T&CMD: T&CM Division
TCM: Traditional Chinese Medicine
TIM: Traditional Indian Medicine
TMM: Traditional Malay Medicine
TPC: Traditional Postnatal Care
UHC: Universal health coverage
WHO: World Health Organization
